# Using the Multidimensional AIMES to Estimate Connection-to-Nature in an Australian Population: A Latent Class Approach to Segmentation

**DOI:** 10.3390/ijerph191912307

**Published:** 2022-09-28

**Authors:** Bradley S. Jorgensen, Julia Meis-Harris

**Affiliations:** 1 Business School, La Trobe University, Bundoora, VIC 3086, Australia; 2 BehaviourWorks Australia, Monash Sustainable Development Institute, Monash University, Clayton, VIC 3800, Australia

**Keywords:** connection to nature, segmentation, latent class analysis

## Abstract

Individuals can interact and develop multiple connections to nature (CN) which have different meanings and reflect different beliefs, emotions, and values. Human population are not homogenous groups and often generalised approaches are not effective in increasing connectedness to nature. Instead, target-group specific approaches focusing on different segments of the population can offer a promising approach for engaging the public in pro-environmental behaviours. This research employed latent class analysis to identify subgroups of individuals in a large, representative sample (*n* = 3090) of an Australian region. Three groups were identified using the AIMES measure of CN with its focus on five types of connection to nature. The high CN group comprised about one-third (35.4%) of participants while the group with the lowest profile of scores contained around a fifth (18.6%) of participants. The majority (46.0%) of participants registered CN levels between the high and low groups. These classes were then regressed on predictor variables to further understand differences between the groups. The largest, consistent predictors of class membership were biocentric and social-altruistic value orientations, stronger intentions to perform pro-environmental behaviours in public (e.g., travel on public transport), the amount of time spent in nature, and the age of participants.

## 1. Introduction

Increasing interactions and connection with nature have been a priority for many government agencies because of its positive outcomes for humans and nature [1,2]. Spending time in nature and feeling psychologically connected to nature have been associated with various wellbeing outcomes, such as positive affect, vitality, and life satisfaction [3,4]. Importantly, it has also been linked to increased engagement in pro-environmental behaviours leading to biodiversity protection [5,6]. There is however still much to learn on how to best foster human-nature connection [7,8]. 

The urban public is not a homogenous group and often generalised approaches are not effective in increasing connectedness to nature. Instead, target-group specific approaches focusing on different segments of the population appear to be promising in engaging the public in pro-environmental behaviours [9,10]. Segmenting a population can help to develop more effective strategies and meet the needs of different communities [9]. The current research segments the Victorian population (Australia) along five dimensions of the AIMES connectedness to nature scale [11]. Findings reveal three distinct types of connection and provide opportunities to develop targeted interventions.

### 1.1. The Multidimensional AIMES Connection to Nature Scale

Building on the findings about connection-to-nature (CN) components over the last two decades, Meis-Harris, Borg and Jorgensen [11] developed and validated a multidimensional measure of CN in cooperation with practitioners and academics working in the field of environmental management and human-nature interactions. The authors named the scale “the AIMES connectedness to nature scale” because it was designed to measure five types of human-nature connections: Attachment, Identity, Material Consumption, Experiential Evaluation, and Spirituality. Confirmatory factor analysis reinforced the five-factor model and showed that all factors were correlated but were statistically distinct from one another. Multi-dimensional measures of CN seek to distinguish different types of nature connection and have better theoretical validity than simpler unidimensional ones [11,12].

The AIMES is based on Ives, et al. [13] theoretical model of CN that highlights multiple ways of connecting to nature: material, experiential, cognitive, emotional, and philosophical. Recent work by Baird, et al. [14] successfully demonstrated the use of this framework by showing how outdoor based nature programs influence the five connection types identified by Ives, Abson, von Wehrden, Dorninger, Klaniecki and Fischer [13]. Furthermore, Riechers, et al. [15] found this multi-dimensional conceptualisation useful in describing the effects of landscape change on CN.

The Attachment dimension describes positive and negative feelings toward nature and entails elements of Perkins [16] Love and Care for Nature scale. Identity relates to a sense of self that is defined in relation to the natural environment focusing on the concept of environmental identity [17,18]. Material consumption can be regarded as valuing nature for the purpose of consuming material goods and services such as food, water, minerals [19,20]. Experiential Evaluation addresses appraisal of direct interaction with natural environments [21] and refers to enjoyment of spending time in nature. Lastly, Spirituality speaks to the feeling of being at one with nature through a belief that all things in nature, including humans, are connected (e.g., [22]).

### 1.2. The Benefits of Segmentation for Target-Group Specific Engagement Approaches

The AIMES scale showed that people differ along the five dimensions. That means the Victorian population is not a homogenous group and therefore one-size-fits all approaches to increase connectedness to nature seem less effective. Instead dividing the Victorian population along the five dimensions of CN may result in more detailed information about the different styles in which Victorians express their connectedness to nature. This segmentation can be used to develop more effective strategies that more specifically meet the needs of the different parties as research has shown that policies are more likely to be accepted when they are designed to fit around individuals’ beliefs and lifestyles [9,23]. Slater [24] describes the aim of segmentation to identify subgroups in the population that cluster together based on their shared values and beliefs. Members of each group or segment are being more similar to each other than members of other groups or segments [24]. 

Segmentation has been widely applied in environmental management to maximise the efforts of communication and engagement strategies. A number of models have focused on major environmental topics such as climate change [25,26,27,28,29,30], sustainability [31], consumption [32,33,34], and conservation [35]. Some other models had a more distinct focus on the human-nature relationship [10,36,37] and environmental worldviews [9] but lack the depth of knowledge that comes when working with a multidimensional approach. 

The current research employs latent class analysis to identify subgroups of individuals that are similar within groups and different between groups. Group formation is based on the items of the AIMES with its focus on five types of connection to nature. These subgroups are then regressed on key variables from the environmental social science literature to further understand differences between the groups. These objectives are reflected in the following research questions:

1. Based on the AIMES, how are latent classes defined to represent individual connections to nature?
2. What is the relationship between CN subgroup membership and key environmental variables (i.e., environmental values, time spent in nature, types of places of connection, types of activities undertaken in nature, pro-environmental intentions and behaviours) and socio-demographic characteristics.

### 1.3. The Study Context

Victoria is Australia’s second most populated State with a population of over 6.6 million. Over the last twenty years, Victoria has had a significant biodiversity loss, leading to the extinction of more than 50 animals and over 50 plant species [2]. Consequently, engaging people to connect to nature and to protect biodiversity is a major aim of the State’s Biodiversity strategy [2]. 

This study thus contributes to the literature, but equally, findings can help to develop targeted interventions that more directly align with the specific sub-groups of how people connect to nature, which may lead to greater connection and pro-conservation behaviours.

## 2. Methods

Participants and sampling. We conducted an online survey in the Australian state of Victoria. The Online Research Unit (ORU), an online survey panel company, recruited a representative sample of adults (18 years or older). Stratified random sampling was employed to ensure the responding sample reflected the Victorian population in relation to gender, age group, and metro versus regional. Participants received an email invitation stating the length, incentive, and close date of the survey. The survey subject was not included to avoid sample selection biases. Email invitations were distributed to 30,753 survey panel members, with a response rate of 9.95%. For more information about data recruitment and survey development see Meis-Harris, Borg and Jorgensen [11] and the State’s Biodiversity strategy [2].

The final sample consisted of *n* = 3090 participants ranging in age from 18 to 89 years. In line with population data from the Australian Bureau of Statistics (ABS) (National, state and territory population, March 2021. Australian Bureau of Statistics, 16 September 2021. Archived from the original on 18 September 2021. Retrieved 26 October 2021.) 50.2% of the sample identified as female (ABS: 50.9%), 23.9% lived in regional Victoria (ABS: 24.5%), and the mean age of respondents was 47 years (SD = 16.31) which is higher than the population (median = 37) as the sample did not include those aged under 18 years. This research was approved by the authors’ University Human Research Ethics Committee (#14010).

To determine the number of classes, four Latent Class Analysis (LCA) models were estimated using MPlus 8. Following Asparouhov and Muthén [38] each model was run using different sets of starting values to ascertain if the loglikelihood was replicated in the bootstrap draws. The Lo-Mendell-Rubin (LMR) test and the bootstrapped likelihood test were used to identify the correct number of classes. A range of goodness-of-fit indices were also consulted to identify the best model. To add to the validity of the results, the statis-tical analysis was performed in two subsamples following a random split of the full sample.

## 3. Results

### 3.1. How Are Latent Classes Defined to Represent Individual Connections to Nature?

The results from the first subsample indicated that the model specifying two classes was preferred (see Table 1). Both the LMR and Vuong-Lo-Mendell-Rubin (VLMR) tests were significant, indicating that two classes were preferred to just one class. These same tests were not significant when three classes were tested, suggesting that the model specifying two classes was again the better model. The remaining goodness-of-fit statistics decrease as more clusters are added. However, the reductions observed in the Model Log Likelihood (LL), Akaike Information Criterion (AIC), Bayesian Information Criterion (BIC) and the Sample-size Adjusted BIC (SABIC) are small for models of three or more classes compared with the reductions observed in these statistics for the model with two classes. Furthermore, the decrease in entropy values over the progression of models is relatively small following the specification of two classes. Finally, the Bootstrap Likelihood Ratio Test (BLRT) was significant for all models such that the addition of more classes results in a better model, but this is likely due to the relatively large sample size serving to increase statistical power and the Type I (see Table 2).

The results of the second subsample suggested that the model with three classes is preferred. In support of the 3-class model, the entropy statistic was highest and the LMR and VLMR tests were significant when three classes were compared with two, but not significant when four classes were compared with three. These results notwithstanding, the Model LL, AIC, BIC and SABIC decreased as more classes were introduced, but these reductions were small following 2-class model.

To choose between the two models, the means of the 20 items were compared between the two and three classes identified in each subsample (see Table 3). These comparisons showed that the classes in each case were ordered rather than nominal. The two class solutions comprised participants whose profile of scores indicated either a high or low connection to nature. Similarly, these classes were ordered as high-, medium-, and low-level connection for the models with three classes. In both subsamples, the third (medium CN) class was formed by splitting both the high and low CN groups from the two-class solution.

**Figure 1:**
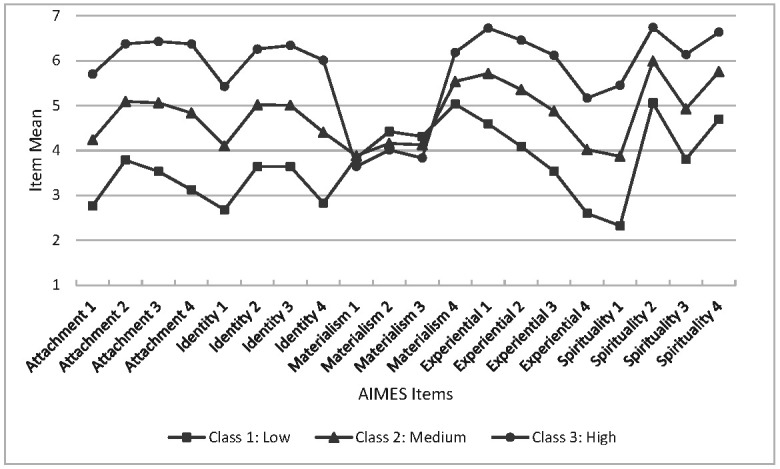
Items means for Subsample 1.

**Figure 2:**
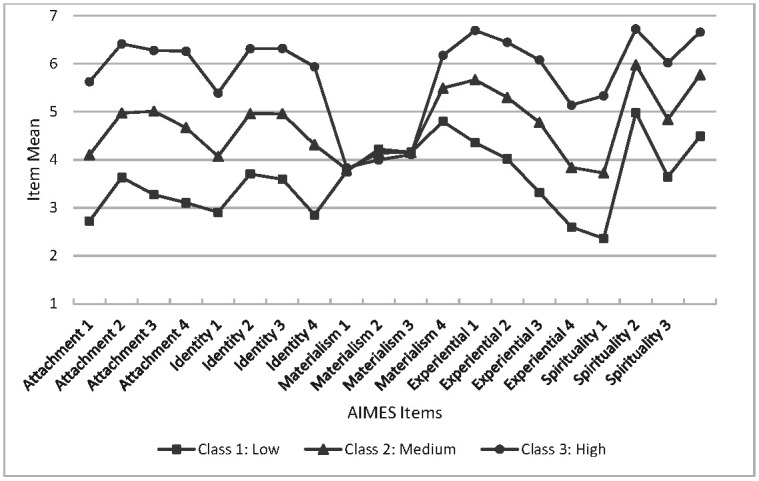
Items mean for Subsample 2.

Three classes were selected for further investigation because the third class was located between the high- and low-CN groups offering greater discrimination between participants on an ordinal metric. The mean scores of each item were plotted to illustrate the high, medium, and low levels of CN that characterise the classes in each subsample (see Figure 1 and Figure 2). First, the pattern of item means is virtually identical in the two random subsamples. Recall that all items were randomly presented to each participant, so the closely matching pattern of item means suggests that the items display considerable consistency between groups. Second, in both subsamples, the first three Materialism items show relatively little discrimination between high/medium/low classes compared with the items measuring other factors. The fourth item has means that resemble the discriminating high, medium, and low pattern observed for the items measuring the Attachment, Identity, Experiential, and Spirituality factors.

Tests were conducted on the item means between subsamples and revealed a similar pattern of results in the two datasets. In subsample 1, all item means were significantly different between classes (*p* < 0.000) except for one Materialism item (Materialism 1) which was not significant (F = 2.63, *p* = 0.073). For the means in the second subsample, the item means were significantly different (*p* < 0.000) except for the means of the three Materialism items noted above (*p* > 0.05 in all three tests).

The two subsamples were pooled and three classes were estimated. As expected, the results for the whole sample were very similar to those reported for the subsamples. The percentage of participants classified into the classes were: highest CN (35.4%), medium CN (46.0%), and low CN (18.6%). The entropy value indexing the classification quality of the model equalled 0.91.

### 3.2. What Is the Relationship between CN Subgroup Membership and Key Environmental Variables and Demographic Characteristics?

Latent Class Analysis (LCA) with auxiliary variables was conducted to identify significant predictors of class membership [39,40]. Having established three classes of participants based on their AIMES scores, the prediction of class membership was conducted using the full sample.

The predictors were those shown in Table 4 and include several demographic characteristics and psychological variables. The information in the table provides a description of each variable and the goodness-of-fit statistics for variables having multiple indicators. Factor scores were employed for multiple indicator variables rather than the latent predictors themselves because of the large computer processing resources such models require [40,41]. For this reason, the reliability coefficients for these variables are also included in the variable descriptions.

Prior to the analysis, zero-order correlations among the predictor variables were examined to identify examples of high collinearity. The largest correlation was between biospheric values and social altruistic values (r = 0.62, *p* < 0.000). The results of the analysis indicated that the correlation between the two value orientations was influencing the sign of the coefficient for the social-altruistic values variable. The coefficient was positive when the biospheric orientation was included in the analysis but negative when it was omitted. For this reason, separate analyses were conducted using either the biospheric variable or the social-altruistic variable.

The results of the categorical latent variables multinomial logistic regressions using the 3-step procedure of Asparouhov and Muthén [40] appear in Table 5 and Table 6. Table 5 presents the results of the equation with biocentric value orientation included as a predictor, while the data in Table 6 contains the social-altruistic value orientation.

From Table 5, increases in the levels of several predictors resulted in decreased odds of being in the low CN cluster compared with the high CN group. That is the odds of reporting a strong CN increased with being older, spending one’s childhood outside Australia, stronger public and private intentions for pro-environmental activities, having spent more time in nature over the last 12 months, and adherence to biospheric and egocentric values. Comparing the medium CN group with individuals classified in the high CN cluster revealed that the odds of membership in the medium CN cluster decreased with higher levels of age, stronger intentions to perform public pro-environmental behaviours, time spent in nature, and stronger support for biospheric values.

The information in Table 6 shows the results of the analysis where the social-altruistic value orientation was substituted for the indicator of biospheric values. Where demographic characteristics were concerned, older participants were more likely to be classified in the high CN group, as were those whose childhood was experienced in a country other than Australia. Furthermore, males were less likely than females to be classified in the low CN class. All remaining variables had significant effects with those in the high CN class more likely to have stronger intentions for pro-environmental activities, spent more time in nature over the last 12 months, and stronger support for biospheric and egocentric values.

Comparing the medium CN group with individuals classified in the high CN cluster, only age emerged as a significant demographic predictor, with older participants less likely to be members of the medium CN class. The odds of membership in the medium CN cluster also decreased with higher levels of pro-environmental intentions, time spent in nature, and greater endorsement of social-altruistic values.

## 4. Discussion

Based on the individual items of the AIMES, three latent classes were found to represent individual connections to nature. These classes represented ordered categories of CN ranging from low to high degrees of individual connections to nature. Class membership was consistently associated with age, willingness to engage in pro-environmental activities in public, spending time in nature, and support for biospheric and social-altruistic values. These results and their implications are discussed in the following sections.

### 4.1. Generalisation from the Sample to the Population

This study employed the AIMES measure of CN to classify a large representative sample into three groups. These groups were ordered on a continuum ranging from lower to higher levels of CN. The high CN group comprised about one-third (35.4%) of participants while the group with the lowest profile of scores on the AIMES items contained around a fifth (18.6%) of participants. The majority (46.0%) of participants registered CN levels between the high and low groups.

When generalised to the population of adults in Victoria (Australian Bureau of Statistics, 2016) (National, state and territory population, March 2021. Australian Bureau of Statistics, 16 September 2021. Archived from the original on 18 September 2021. Retrieved 26 October 2021) approximately 1.6 million Victorian adults are likely to experience a relatively strong connection to nature. On the other hand, about 900,000 adults have relatively little connection. The large class of participants defined by medium levels of CN (about 2 million adults), as well as the smaller yet still substantial proportion of participants classified as low CN, suggests there is considerable scope to improve the range and quality of Victorians’ connections to nature and, therefore, the wellbeing they might derive from these connections and the benefits to biodiversity [3,5,6].

No matter what the CN group, beliefs about the material consumption of nature were not a defining characteristic in terms of membership. This is unsurprising given that the Material Consumption factor of the AIMES is largely independent of the other factors [11,43]. Therefore, some individuals held stronger materialism beliefs across levels of CN. Within the adult Victorian population, individuals do not make sense of material connections to nature in the same way they experience connections of identity, affect, experience in nature, or spirituality [44,45,46].

One might suppose that the anthropocentric underpinnings of beliefs in the primacy of the material goods and services supplied by nature would stand in direct contrast to the more biospheric orientations of CN. For example, other work with the AIMES has shown that the Material Consumption dimension was significantly correlated with egocentric values and positively correlated with biospheric and social-altruistic values [11]. However, instead of observing high levels of materialism contributing to the formation of low levels of CN, the data indicate that individuals do not necessarily bring into relationship the exploitation of nature to satisfy their material consumption needs with their spiritual, emotional, and identity connections. In other words, a connection to nature expressed through an appreciation of its contribution to meeting material needs, over and above its intrinsic value, is a relationship fundamentally different to the identity, attachment, experiential, and spiritual connections examined here and in other CN research [1]. Given that so much of nature is exploited for the purpose of facilitating and encouraging a material connection to nature via consumption of goods and services, pro-conservation interests might renew efforts to communicate the consequences of material consumption for threatened ecosystems and its sustainable limits [47,48].

### 4.2. Consistent Predictors of Class Membership

Comparison of the low CN and medium CN classes with the high CN class revealed a subset of predictor variables that are consistently significant in predicting CN group membership. Participants’ age was the only demographic variable that was significant across all regressions showing that those in the two lower CN groups were likely to be younger than members of the highest-scoring group on the AIMES. Furthermore, participants in the low and medium CN groups were less likely to report public pro-environmental intentions, spent less time in nature, and were less supportive of both biospheric and social-altruistic value orientations than those in the highest CN group.

Of the remaining variables in the analyses, pro-environmental intentions to perform behaviours in private contexts was a significant predictor in all regressions but the one comparing the medium and high CN classes. This is evidence suggesting that the biospheric value orientation explains the relationship between intentions and membership in these classes whereas social-altruistic values do not.

Other predictors showed a pattern of relationships with CN class membership that appeared to be independent of whether biospheric or social-altruistic value orientation was include in the regression analysis. For example, support for egoistic values had a small-to moderate predictive effect only when the lowest level CN class was compared with the highest. Egoistic value orientation was not a significant predictor of membership in the medium CN class compared with the high CN group. Similarly, whether participants spent their childhood outside Australia or within it, and whether they identified as male or female, depended to an extent on which CN classes constituted the dependent variable. Regression coefficients tended to be larger when membership in the low and high CN classes was the focus of analysis rather than membership in the medium and high groups.

### 4.3. Value Orientations and CN Class Membership

That social-altruistic and biospheric values were held by individuals reporting strong connections to nature is consistent with previous research [49]. The observation that holding an egocentric value orientation can also support high levels of connection is not widely reported in the literature but is unsurprising upon reflection.

Previous research by Bouman, et al. [50] reported significant positive correlations between self-reports of how frequently participants showered/bathed in an average week and/or bath a week (an energy conservation behaviour) and both egocentric and biospheric value orientations. Imaningsih, et al. [51] examined the effect of egocentric and biospheric values (among other variables) on outcomes such as consumers’ purchasing loyalty to green products. The researchers found that both egoistic and biospheric values had positive effects on green loyalty.

In related research, Hansla, et al. [52] reported significant positive correlations among environmental concern for oneself, others and the biosphere and three-out-of-four values (i.e., achievement, benevolence, and universalism). The fourth value–power–was positively related to environmental concern for oneself, but not to either concern for others or the biosphere.

The aforementioned studies, while examples of contexts in which egocentric and biospheric value positions appear to support pro-environmental behaviour and concern for the environment, values research has tended to report that biospheric and egocentric orientations operate counter to each other when predicting connection to nature and pro-environmental outcomes generally (e.g., [49,50,51,52,53]).

Evidence for this counter relationship notwithstanding, interventions have engaged egocentric beliefs and values to promote pro-environmental behaviour change. For example, environmental campaigns have sought to reduce energy and water consumption by pointing to the cost savings accruing from resource conservation. Further, the protection of endangered species is underpinned by the opportunity to continue experiencing them first-hand as much as the importance of the biodiversity of ecological systems. Environmental behaviours can be underpinned by multiple motives that span all three value orientations.

An earlier construct validity analysis of the AIMES [11] showed that egocentric values were statistically unrelated to all AIMES dimensions except Materialism. A relationship between egocentricism and connection to nature via its material consumption is consistent with a good deal of thinking in environmental behaviour research [46,54,55] and sustainable consumption [47]. Baird, Dale, Holzer, Hutson, Ives and Plummer [14] for example refer to materialism as representing a “shallow connections to nature” (p. 3) recognising its basis in anthropocentrism and distinguishing it from “deeper” forms of connection such as cognitive and emotional connections.

Recall that the Material consumption dimension of the AIMES was not a strong contributor to the formation of the CN classes and the classification of participants. Therefore, the effect of egocentric values that might counter the effects of biospheric and social-altruistic value orientations was diminished or negated. Without the influence of material consumption in the formation of the classes, the effect of egocentric values that did emerge supported, rather than contradicted, the effects of the other two value orientations.

While this explanation is speculative at this stage, future research might attempt to focus on how the relationships between value positions can influence environmental variables of interest. A study by de Groot and Steg [56] begun research along these lines and found that conflict between altruistic and biospheric goals provided a unique source of influence on pro-environmental intentions. Future research along these lines may provide insights into not only how the level of support for a particular value position can affect behaviour, but also how its relationship with other value orientation may offer a distinct motivational basis.

## 5. Conclusions

The AIMES measure of CN was used to classify a large representative sample into three latent classes which were ordered on a continuum ranging from lower to higher levels of CN. Beliefs about the primacy of nature as an input to material goods and their consumption was the only dimension of the AIMES that did not contribute to the formation of the latent classes. This suggests that materialism as measure by the AIMES is a relatively distinct type of connection compared to other types of connections studied in previous CN research.

On the basis of the representative sample employed in the research, it was possible to generalise the sample statistics to the wider population of adults aged 18 years or more. The majority of the population were classified as having medium and high levels of connection to nature, and classification into the different classes was consistently predicted by public pro-environmental intentions, time in nature, and both biospheric and social-altruistic value orientations.

### Future Research

Future research might further explore the validity of the AIMES and multidimensional approaches to CN in general [13]. Riechers, Pătru-Dușe and Balázsi [15], for example, showed that a multidimensional appreciation of CN was required to capture the diversity of effects associated with different types of landscapes and the human relationships associated with them. Baird, Dale, Holzer, Hutson, Ives and Plummer [14] also benefitted from a multidimensional approach when evaluating environmental education programs. Further validation and development of the AIMES provides researchers with the benefits of understanding keyways individuals can connect with nature, and can facilitate a comparison of research results across different research contexts in which CN has been measured by the same instrument.

This study has provided further evidence that individuals do not make sense of material connections to nature in the same way they experience connections of identity, affect, experience in nature, or spirituality. Baird, Dale, Holzer, Hutson, Ives and Plummer [14] have suggested that the five types of connections developed by Ives, Abson, von Wehrden, Dorninger, Klaniecki and Fischer [13] might be conceived as systematically varying from shallow to deeper connections, and that future research might seek to test this position. Our results support this need for future research and suggest that it be examined in the context of different types of pro-environmental behaviours (e.g., dematerialisation behaviours) varying in the level of commitment required to perform them and involve subpopulations that are likely to prioritise different connections to nature (e.g., pro-environmental versus pro-development groups).

## Figures and Tables

**Table 1:** Determination of class number for Group 1–20 items.

Class	Model LL	AIC	BIC	SABIC	Entropy	Smallest Class (%)	LMR (*p*)	VLMR (*p*)	BLRT (*p*)
1	−55,224.43	110,528.86	110,742.57	110,615.50	-	-	-	-	-
2	−50,411.21	100,944.43	101,270.34	101,076.55	0.930	43.56	0.000	0.000	0.000
3	−48,955.12	98,074.23	98,512.34	98,251.85	0.906	22.01	0.377	0.375	0.000
4	−48,157.69	96,521.38	97,071.68	96,744.48	0.914	7.83	0.132	0.131	0.000
5	−47,854.37	95,956.73	96,619.24	96,225.32	0.909	3.11	0.231	0.229	0.000

**Table 2:** Determination of class number for Group 2–20 items.

Class	Model LL	AIC	BIC	SABIC	Entropy	Smallest Class (%)	LMR (*p*)	VLMR (*p*)	BLRT (*p*)
1	−55,086.17	110,252.34	110,466.05	110,338.98	-	-	-	-	-
2	−50,758.43	101,638.86	101,964.77	101,770.98	0.915	49.06	0.000	0.000	0.000
3	−49,229.68	98,623.36	99,061.47	98,800.98	0.917	16.96	0.006	0.006	0.000
4	−48,501.56	97,209.12	97,759.43	97,432.22	0.911	5.31	0.303	0.301	0.000
5	−48,220.45	96,688.91	97,351.41	96,957.49	0.875	3.75	0.210	0.210	0.000

**Table 3:** Distribution across the classes for two and three classes for subsample 1 and 2.

Subsample	Class 1 (High)	Class 2 (Low)	Class 3 (Medium)
	*n* (%)	*n* (%)	*n* (%)
1	872 (56.4)	673 (43.6)	-
1	516 (33.4)	340 (22.0)	689 (44.6)
2	787 (50.9)	758 (49.1)	-
2	545 (35.3)	262 (16.9)	738 (47.8)

**Table 4:** Variable description and goodness-of-fit.

Variable	Description		Goodness-of-Fit
Age	Please specify your age: _______ years Responses were post-coded into the following categories:		-
	18–24	50–54	
	25–29	55–59	
	30–34	60–64	
	35–39	65–69	
	40–44	70–74	
	45–49	75 plus	
Gender	Please specify your gender: 1. Female 2. Male 3. Other (specify) ^a^		-
Childhood in Australia	Did you spend any of your childhood living in Australia? 1. Yes 2. No		-
Pro-environmental Intentions	The likelihood of undertaking 11 public (e.g., “volunteering in community-based activities”) and private (e.g., “reducing energy use”) activities over the next 12 months were measured on 7-point scales. The construct reliabilities equalled 0.87 and 0.68, respectively.		χ^2^ (*df*) = 382.98 (31) *p <* 0.05; CFI = 0.98; RMSEA = 0.06; SRMR = 0.04
Time Spent in Nature	Measured with a single item: “In the last year, about how often have you generally spent time in nature?” Response options were 1 *(never)*; 2 *(less than once a year)*; 3 *(at least once a year)*; 4 *(at least twice a year)*; 5 *(at least once a month)*; 6 *(at least once a fortnight)*; 7 *(at least once a week)*; 8 *(every other day)*; and 9 *(every day)*.		-
Values Orientations	Biospheric (α = 0.91), Social-Altruistic (α = 0.82), and Egocentric (α = 0.72) value orientations were measured following Stern, et al. [42].		χ^2^ (*df*) = 394.16 (49) *p < 0*.05; CFI = 0.97; RMSEA = 0.07; SRMR = 0.05

^a^ Just two participants selected the “other” response option and were excluded from the analysis.

**Table 5:** Comparison of membership between classes with Biocentric Value Orientation included.

Variable	Estimate	S.E.	Est./S.E.	*p*-Value
Class 1 (Low CN) compared to Class 3 (High CN)				
1. Intercept	6.258	0.566	11.055	0.000
2. Age	−0.171	0.027	−6.274	0.000
3. Gender	−0.294	0.170	−1.725	0.085
4. Australian Childhood	−0.512	0.211	−2.421	0.015
5. Public pro-environmental intentions	−1.299	0.136	−9.577	0.000
6. Private pro-environmental intentions	−0.456	0.159	−2.869	0.004
7. Time spent in nature	−0.782	0.055	−14.097	0.000
8. Biospheric value orientation	−3.010	0.153	−19.715	0.000
9. Egocentric value orientation	−0.289	0.114	−2.537	0.011
Class 2 (Medium CN) compared to Class 3 (High CN)				
1. Intercept	4.433	0.404	10.978	0.000
2. Age	−0.073	0.018	−4.062	0.000
3. Gender	−0.059	0.114	−0.515	0.607
4. Australian Childhood	−0.138	0.134	−1.027	0.304
5. Public pro-environmental intentions	−0.549	0.089	−6.182	0.000
6. Private pro-environmental intentions	−0.051	0.114	−1.322	0.186
7. Time spent in nature	−0.385	0.039	−9.891	0.000
8. Biospheric value orientation	−1.789	0.101	−17.793	0.000
9. Egocentric value orientation	−0.128	0.069	−1.860	0.063

**Table 6:** Comparison of membership between classes with Social-Altruistic Value Orientation included.

Variable	Estimate	S.E.	Est./S.E.	*p*-Value
Class 1 (Low CN) compared to Class 3 (High CN)				
1. Intercept	5.889	0.499	11.795	0.000
2. Age	−0.208	0.024	−8.497	0.000
3. Gender	−0.310	0.151	−2.054	0.040
4. Australian Childhood	−0.455	0.181	−2.510	0.012
5. Public pro-environmental intentions	−1.165	0.120	−9.722	0.000
6. Private pro-environmental intentions	1.034	0.143	−7.220	0.000
7. Time spent in nature	−0.671	0.048	−14.017	0.000
8. Social-Altruistic value orientation	−1.335	0.108	−12.317	0.000
9. Egocentric value orientation	−0.277	0.096	−2.885	0.004
Class 2 (Medium CN) compared to Class 3 (High CN)				
1. Intercept	3.866	0.362	10.673	0.000
2. Age	−0.082	0.016	−5.092	0.000
3. Gender	−0.025	0.104	−0.241	0.809
4. Australian Childhood	−0.152	0.120	−1.275	0.202
5. Public pro-environmental intentions	−0.526	0.079	−6.651	0.000
6. Private pro-environmental intentions	−0.439	0.111	−3.945	0.000
7. Time spent in nature	−0.346	0.036	−9.710	0.000
8. Social-Altruistic value orientation	−0.927	0.084	−10.980	0.000
9. Egocentric value orientation	−0.079	0.063	−1.249	0.212

## Data Availability

Data available at Open Science Framework: Data from: Victorians Valuing Nature Foundations Survey.

## References

[1] Restall B., Conrad E. (2015). A literature review of connectedness to nature and its potential for environmental management. J. Environ. Manag..

[2] DELWP (2017). Protecting Victoria’s Environment—Biodiversity 2037.

[3] Martin L., White M.P., Hunt A., Richardson M., Pahl S., Burt J. (2020). Nature contact, nature connectedness and associations with health, wellbeing and pro-environmental behaviours. J. Environ. Psychol..

[4] Capaldi C.A., Dopko R.L., Zelenski J.M. (2014). The relationship between nature connectedness and happiness: A meta-analysis. Front. Psychol..

[5] Mackay C.M.L., Schmitt M.T. (2019). Do people who feel connected to nature do more to protect it? A meta-analysis. J. Environ. Psychol..

[6] Whitburn J., Linklater W., Abrahamse W. (2019). Meta-analysis of human connection to nature and proenvironmental behavior. Conserv. Biol..

[7] Lumber R., Richardson M., Sheffield D. (2017). Beyond knowing nature: Contact, emotion, compassion, meaning, and beauty are pathways to nature connection. PLoS ONE.

[8] Bruni C.M., Schultz P.W. (2010). Implicit beliefs about self and nature: Evidence from an IAT game. J. Environ. Psychol..

[9] Bernstein J., Szuster B. (2018). Beyond unidimensionality: Segmenting contemporary pro-environmental worldviews through surveys and repertory grid analysis. Environ. Commun..

[10] Gkargkavouzi A., Paraskevopoulos S., Matsiori S. (2018). Who cares about the environment?. J. Hum. Behav. Soc. Environ..

[11] Meis-Harris J., Borg K., Jorgensen B.S. (2021). The construct validity of the multidimensional AIMES connection to nature scale: Measuring human relationships with nature. J. Environ. Manag..

[12] Tam K.-P. (2013). Concepts and measures related to connection to nature: Similarities and differences. J. Environ. Psychol..

[13] Ives C.D., Abson D.J., von Wehrden H., Dorninger C., Klaniecki K., Fischer J. (2018). Reconnecting with nature for sustainability. Sustain. Sci..

[14] Baird J., Dale G., Holzer J.M., Hutson G., Ives C.D., Plummer R. (2022). The role of a nature-based program in fostering multiple connections to nature. Sustain. Sci..

[15] Riechers M., Pătru-Dușe I.A., Balázsi Á. (2021). Leverage points to foster human–nature connectedness in cultural landscapes. Ambio.

[16] Perkins H.E. (2010). Measuring love and care for nature. J. Environ. Psychol..

[17] Clayton S.D., Clayton S.D., Opotow S. (2003). Environmental identity: A conceptual and an operational definition. Identity and the Natural Environment.

[18] Schultz P.W., Schmuck P., Schultz W.P. (2002). Inclusion with nature: The psychology of human-nature relations. Psychology of Sustainable Development.

[19] Kendal D., Ford R.M., Anderson N.M., Farrar A. (2015). The VALS: A new tool to measure people’s general valued attributes of landscapes. J. Environ. Manag..

[20] Winter C., Lockwood M. (2004). The natural area value scale: A new instrument for measuring natural area values. Australas. J. Environ. Manag..

[21] Nisbet E.K., Zelenski J.M., Murphy S.A. (2009). The nature relatedness scale: Linking individuals’ connection with nature to environmental concern and behavior. Environ. Behav..

[22] Garfield A.M., Drwecki B.B., Moore C.F., Kortenkamp K.V., Gracz M.D. (2014). The oneness beliefs scale: Connecting spirituality with pro-environmental behavior. J. Sci. Study Relig..

[23] Corner A., Randall A. (2011). Selling climate change? The limitations of social marketing as a strategy for climate change public engagement. Glob. Environ. Chang..

[24] Slater M.D. (1996). Theory and method in health audience segmentation. J. Health Commun..

[25] Maibach E.W., Leiserowitz A., Roser-Renouf C., Mertz C. (2011). Identifying like-minded audiences for global warming public engagement campaigns: An audience segmentation analysis and tool development. PLoS ONE.

[26] Hall M.P., Lewis Jr N.A., Ellsworth P.C. (2018). Believing in climate change, but not behaving sustainably: Evidence from a one-year longitudinal study. J. Environ. Psychol..

[27] Morrison M., Hine D.W., Phillips W.J., Driver A.B., Morrison M. (2017). Audience segmentation and climate change communication. Oxford Research Encyclopedia of Climate Science.

[28] Barnes A.P., Toma L. (2012). A typology of dairy farmer perceptions towards climate change. Clim. Chang..

[29] Yee S.H., Paulukonis E., Simmons C., Russell M., Fulford R., Harwell L., Smith L. (2021). Projecting effects of land use change on human well-being through changes in ecosystem services. Ecol. Model..

[30] Asah S.T., Blahna D.J., Ryan C.M. (2012). Involving Forest Communities in Identifying and Constructing Ecosystem Services: Millennium Assessment and Place Specificity. J. For..

[31] Poortinga W., Darnton A. (2016). Segmenting for sustainability: The development of a sustainability segmentation model from a Welsh sample. J. Environ. Psychol..

[32] Balderjahn I., Peyer M., Seegebarth B., Wiedmann K.-P., Weber A. (2018). The many faces of sustainability-conscious consumers: A category-independent typology. J. Bus. Res..

[33] Dickson M.A. (2001). Utility of No Sweat Labels for Apparel Consumers: Profiling Label Users and Predicting Their Purchases. J. Consum. Aff..

[34] Verain M.C.D., Bartels J., Dagevos H., Sijtsema S.J., Onwezen M.C., Antonides G. (2012). Segments of sustainable food consumers: A literature review. Int. J. Consum. Stud..

[35] MacDonald E., Harbrow M., Jack S., Kidd J., Wright A., Tuinder P., Balanovic J., Medvecky F., Poutasi M. (2019). Segmenting urban populations for greater conservation gains: A new approach targeting cobenefits is required. Conserv. Sci. Pract..

[36] Kahn P.H., Lourenço O. (2002). Water, Air, Fire, and Earth:A Developmental Study in Portugal of Environmental Moral Reasoning. Environ. Behav..

[37] Marais-Potgieter A., Thatcher A. (2020). Identification of Six Emergent Types Based on Cognitive and Affective Constructs that Explain Individuals’ Relationship with the Biosphere. Sustainability.

[38] Asparouhov T., Muthén B. (2012). Using Mplus TECH11 and TECH14 to test the number of latent classes. Mplus Web Notes.

[39] Vermunt J.K. (2010). Latent class modeling with covariates: Two improved three-step approaches. Political Anal..

[40] Asparouhov T., Muthén B. (2014). Auxiliary Variables in Mixture Modeling: Three-Step Approaches Using Mplus. Struct. Equ. Modeling A Multidiscip. J..

[41] Ferguson S.L., G. Moore E.W., Hull D.M. (2020). Finding latent groups in observed data: A primer on latent profile analysis in Mplus for applied researchers. Int. J. Behav. Dev..

[42] Stern P.C., Dietz T., Guagnano G.A. (1998). A brief inventory of values. Educ. Psychol. Meas..

[43] Meis-Harris J., Saeri A., Boulet M., Borg K., Faulkner N., Jorgensen B. (2019). Victorians Value Nature: Survey Results.

[44] Brewer G.D., Stern P.C. (2005). Decision Making for the Environment: Social and Behavioral Science Research Priorities.

[45] Hirsh J.B., Dolderman D. (2007). Personality predictors of Consumerism and Environmentalism: A preliminary study. Personal. Individ. Differ..

[46] Kilbourne W., Pickett G. (2008). How materialism affects environmental beliefs, concern, and environmentally responsible behavior. J. Bus. Res..

[47] Jackson T. (2005). Live better by consuming less?: Is there a “double dividend” in sustainable consumption?. J. Ind. Ecol..

[48] Whitmarsh L., Capstick S., Nash N. (2017). Who is reducing their material consumption and why? A cross-cultural analysis of dematerialization behaviours. Philos. Trans. R. Soc. A Math. Phys. Eng. Sci..

[49] Aguilar-Luzón M.C., Carmona B., Calvo-Salguero A., Castillo Valdivieso P.A. (2020). Values, environmental beliefs, and connection with nature as predictive factors of the pro-environmental vote in Spain. Front. Psychol..

[50] Bouman T., Steg L., Kiers H.A. (2018). Measuring values in environmental research: A test of an environmental portrait value questionnaire. Front. Psychol..

[51] Imaningsih E.S., Tjiptoherijanto P., Heruwasto I., Aruan D.T.H. (2019). Linking of egoistic, altruistic, and biospheric values to green loyalty: The role of green functional benefit, green monetary cost and green satisfaction. J. Asian Financ. Econ. Bus..

[52] Hansla A., Gamble A., Juliusson A., Gärling T. (2008). The relationships between awareness of consequences, environmental concern, and value orientations. J. Environ. Psychol..

[53] Schultz P.W., Shriver C., Tabanico J.J., Khazian A.M. (2004). Implicit connections with nature. J. Environ. Psychol..

[54] Banerjee B., McKeage K. (1994). How green is my value: Exploring the relationship between environmentalism and materialism. NA-Advances in Consumer Research.

[55] Polonsky M., Kilbourne W., Vocino A. (2014). Relationship between the dominant social paradigm, materialism and environmental behaviours in four Asian economies. Eur. J. Market..

[56] de Groot J.I., Steg L. (2007). Value orientations and environmental beliefs in five countries: Validity of an instrument to measure egoistic, altruistic and biospheric value orientations. J. Cross-Cult. Psychol..

